# Establishing a Dementia-Inclusive Community: The Evaluation of Dementia Friends in Cleveland Heights

**DOI:** 10.1093/geroni/igab046.1983

**Published:** 2021-12-17

**Authors:** Jessica Bibbo, Jennifer Cardellini, Sarah Nicolay

**Affiliations:** Benjamin Rose Institute on Aging, Cleveland, Ohio, United States

## Abstract

Cleveland Heights, in northeast Ohio, is currently working towards becoming a member of the Dementia Friendly America National Network. Utilizing the Dementia Friends curriculum to raise community members’ awareness of issues related to dementia is a key component of this initiative. Our initial efforts toward this goal targeted two sectors, namely community member and libraries. Participants completed on-line surveys at the beginning and end of each session. The surveys include the Brief Tool for Dementia-Friendly Education and Training Sessions developed by the Administration for Community Living. Of the 22 participants, nine had not previously attended a Dementia Friends session and completed both pre- and post-session surveys. Results indicated participants felt more confident interacting with people living with dementia at post-session compared to pre-session (t = -2.83, p=.022). Changes at the individual level may create more inclusive communities for people living with dementia and those who care for and about them.

